# Experimental variables in sugarcane intercropping in Reunion Island for data matching

**DOI:** 10.1016/j.dib.2022.108869

**Published:** 2022-12-31

**Authors:** Sandrine Auzoux, Billy Ngaba, Mathias Christina, Benjamin Heuclin, Mathieu Roche

**Affiliations:** a UR AIDA (Agroecology and sustainable intensification of annual crops), University of Montpellier, CIRAD, La Réunion, France; b UMR TETIS (Land, Environment, Remote Sensing and Spatial Information), University of Montpellier, AgroParisTech, CIRAD, CNRS, INRAE, Montpellier, France; c French Agricultural Research for Development (CIRAD), France

**Keywords:** Companion plant, Data integration, Data reconciliation, Text mining

## Abstract

This study aimed to link experimental data dealing with complex agroecological systems. For sharing and linking collected data with the generic AEGIS (Agro-Ecological Global Information System) database, our work described in this data paper consists in mapping researcher variables to the AEGIS dictionary variable for different tropical crops (sugarcane, rice, sorghum or cover crops). Additionally, this data paper presents a study case based on sugarcane intercropping systems for evaluating 3 matching measures of variables.

**Specifications Table**

**Table:** 

Subject	Agronomy and Crop Science, Data Science
Specific subject area	Cropping systems of sugar cane in association with cover crops.
Type of data	Texts.
How data were acquired	Primary source: (i) List of experimental variable names acquired manually by a community of researchers in a network of trials performed from 1987 to 2022 in La Réunion, Madagascar, Mali, Senegal and Burkina Faso [list_of_researcher_variables.txt]; (ii) List of standardized variables names obtained from the AEGIS information system variable dictionary [list_of_candidate_variables_AEGIS.txt]. Secondary source: (iii) relevant matching between (i) and (ii) obtained manually (i.e. ground truth) [Correspondances.txt].
Data format	Filtered
Description of data collection	(i) Variable names from agroecological trials described by researchers, (ii) Variable names from the AEGIS variable dictionary, (iii) Matched variables.
Data source location	The data are hosted on the CIRAD Dataverse. The data were collected by CIRAD, La Réunion, France (Latitude: -21.1, Longitude: 55.5).
Data accessibility	Repository name: CIRAD Dataverse Data identification number: https://doi.org/10.18167/DVN1/XDHKR8 Direct URLs to data: https://dataverse.cirad.fr/dataset.xhtml?persistentId=doi:10.18167/DVN1/XDHKR8 [Primary and secondary source] https://dataverse.cirad.fr/dataverse/aida [Primary source] https://dataverse.cirad.fr/dataverse/APEEDAIS [Primary source]

## Value of the Data

• These datasets contribute to the available resources on specialized domains in agriculture and more specifically in agrosystems in rotation or intercropping including cover crops agroecological.
• These datasets can be used by agronomists for normalizing data according to standard attributes of agrosystems.
• These datasets are useful for improving reconciliation methods of agrosystem databases.
• These datasets can be used by computer scientists in order to evaluate text-mining approaches to match attribute names.

## 1 Objective

To address challenges on a global scale such as food safety, reduction of environmental impacts, and climate change, CIRAD adopts agro-ecological approaches to design and evaluate systems that make more efficient use of natural resources and mobilise plant biodiversity. Various trials were performed and each researcher has his own way of naming variables and describing them. Consequently, there is a need to standardize these heterogeneous data. This paper deals with data mapping by researchers that describe cropping systems of sugarcane, rice, sorghum and cotton in association or in rotation with cover crops in different countries (La Réunion, Madagascar, Mali, Senegal and Burkina Faso) [1], [2]. A cover crop is a plant that provide ecosystem services in agrosystems, such as erosion control, soil fertility improvement, pest control, weed control and increasing biodiversity.

CIRAD has developed AEGIS (Agro-ecological Global Information System) [3] to store, manipulate, disseminate and enhance data collected in agro-ecological systems. It integrates a harmonised data acquisition and processing chain using a variable dictionary [4] to describe and ensure the quality and interoperability of the data. A variable consists of semantic terms derived from expert knowledge and reference ontologies. Feedback from stakeholders (researchers, agricultural technicians and engineers) on their data has allowed the variable dictionary to evolve and to establish a list of common variables to facilitate data comparison and analysis, as well as links with crop models.

For mapping collected data with the generic database of AEGIS, the first step consists in structuring and standardizing experimental datasets. The second step consists of mapping researcher variables from experimental datasets to AEGIS variable dictionary. This data paper focuses on this second step of the work.

## 2 Data Description

The list of researcher variables comes from datasets collected on 185 trials performed in the different countries from 1992 to 2021, https://dataverse.cirad.fr/dataverse/aida (primary source). The trials were performed by different researchers in different sites. Each dataset includes variables that describe (i) experimental design, (ii) growth measurements (i.e. biomass, recovery rate) of main crop and cover crops, (iii) observations (scoring, floristic survey) at the scale of each weed species in the plots, (iv) cultural practices and (v) environmental conditions. The list of experimental variable names acquired manually is proposed in our dataset: *list_of_researcher_variables.txt* (primary source).

In order to share, reuse and link these datasets with AEGIS, we have to match researcher variables with variable dictionary. The list of standardized variables names obtained from the AEGIS is given in our dataset: *list_of_candidate_variables_AEGIS.txt* (primary source).

To sum-up, we use two types of data as primary source:

1. *researcher variables* with the following information (see an example in Table 1):
  • variable name,
  • description,
  • unit,
  • class,
  • subclass,
  • domain,
  • studied crop.

**Table 1:** Examples of researcher variables.

Variable name	Description	Unit

Yield_CAS	Cane yield (in fresh machinable stem)	t.ha-1
Sugar_CAS	Sugar content of fresh stem mass	%
IFTH	Herbicide Application Frequency Index	[0.1]
2. *candidate variables* (i.e. AEGIS variable dictionary) with the following information (see an example in Table 2):
  • variable name which is defined from the concatenation of an entity, a trait and a unit of measurement,
  • description,
  • unit,
  • class,
  • subclass,
  • domain.

**Table 2:** Examples of candidate variables.

Variable name	Description	Class	Subclass	Domain
root_crop_yield_dm_t.ha1	measurement of root dry biomass at plot level	experimental variable	plant	agronomy
ferti_K_app_rate_kg.ha1	potassium application rate for soil fertilization	itk	organic fertilization	soil
abv_sugar_fm_content_%	percentage of sugar of the fresh matter overground biomass	experimental variable	plant	biomass quality

A dedicated dataset has been manually constructed by experts (a part of the co-authors of this data paper) to obtain relevant matching between *researcher variables* and *candidate variables* (i.e. ground truth) and is given in the dataset: *Correspondances.txt* (secondary source).

**Fig. 1:**
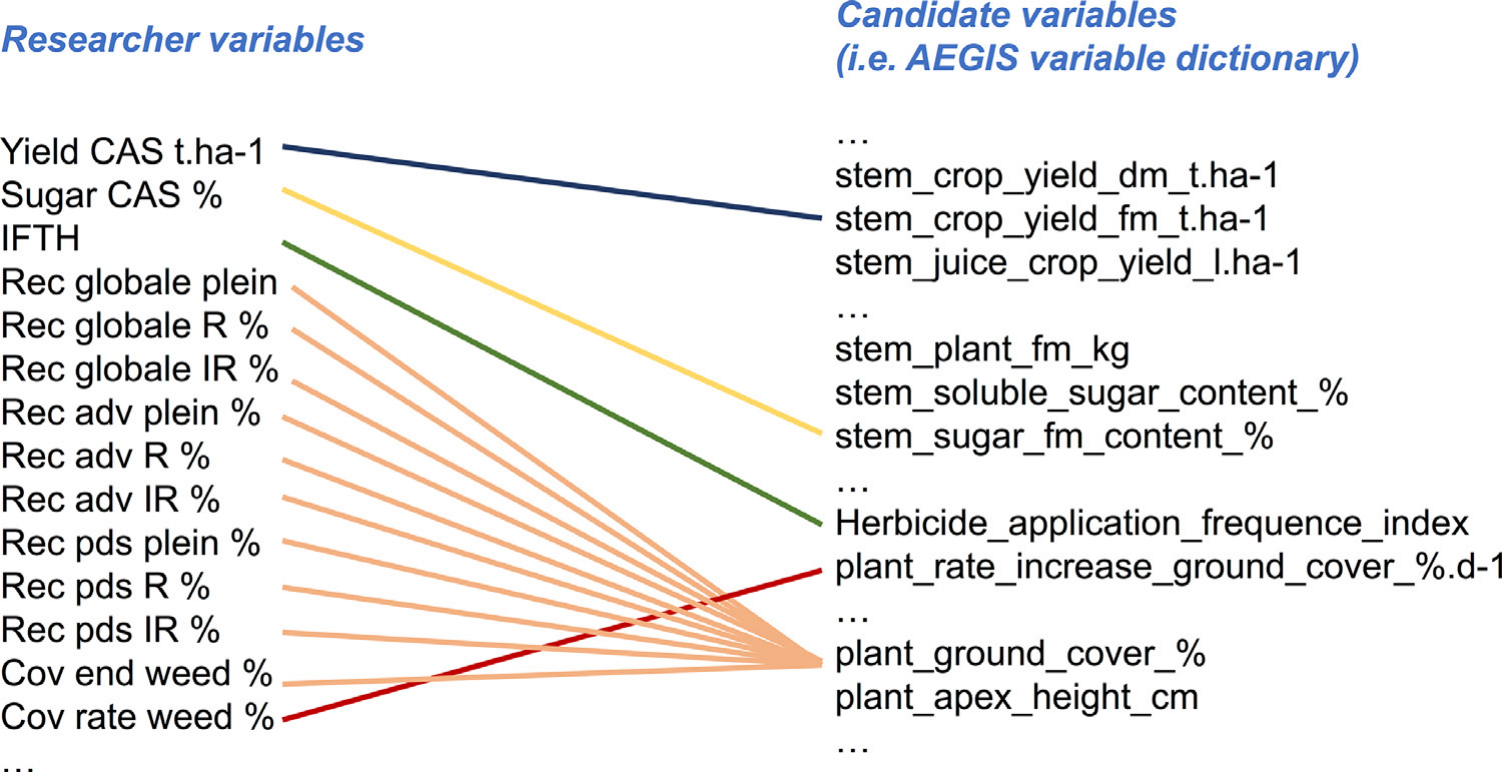
Examples of link between ‘Researcher variables’ and ‘AEGIS variables’.

Examples of matching variables are given in Fig. 1.

To summarise, this experimental dataset consists of 3 files: (i) the list of variables from trials described by researchers, (ii) the list of variables from the AEGIS variable dictionary, (iii) the list of relevant matches between both lists: https://dataverse.cirad.fr/dataset.xhtml?persistentId=doi:10.18167/DVN1/XDHKR8.

A method of automatic matching approach between researcher and candidate variables is described in the following section. This method was applied on a sub-sample of researcher variables (*Correspondances_study_case.txt*) from a network of sugarcane intercropping trials with cover crops (28 datasets from the APEEDAIS dataverse, https://dataverse.cirad.fr/dataverse/APEEDAIS).

## 3 Experimental Design, Materials and Methods

To link “researcher variables” and “candidate variables”, we propose to use text mining and information retrieval methods [5], [6]. We use two main approaches (i.e. Lev and Cos) that can be combined (i.e. Comb):

• **Lexical measure**: The aim of this approach is to compare variable names based on their character string. For this approach, we applied the Levenshtein distance with normalisation [7] (see Formula (1)) which calculates the number of changes between two character strings of the variable names. The Levenhstein distance (i.e., L in Formula (1)) between two strings is given by the minimum number of operations needed to transform one source string (i.e., $s_{1}$ in Formula (1)) into the other string (i.e., $s_{2}$ in Formula (1)), where an operation is an insertion, deletion, or substitution of a single character.
  (1) $$Lev\left(s_{1},s_{2}\right)=\max\left\{0,\frac{\min\{|s_{1}\left|,\right|s_{2}\left|\right\}-L\left(s_{1},s_{2}\right)}{\min\{|s_{1}|,|s_{2}|\}}\right\}$$
• **Contextual measure**: The objective of this approach is to compare the variables based on their description. This description as a “bag of words” representation (i.e. vector space model) is related to textual contexts of each variable [8]. These contexts can be compared with similarity measures like the cosine measure [6] between both vectorized descriptions (i.e. $v_{1}$ and $v_{2}$) (see Formula (2)).
  (2) $$Cos\left(v_{1},v_{2}\right)=\frac{v_{1}\;v_{2}}{∥v_{1}∥∥v_{2}∥}$$
  Some pre-processing approaches like lemmatization processing could be applied. Lemmatization consists in taking into account the base form for each word (e.g. plants → plant, could → can, etc.) in the “bag of words” representation.
• **Combined measure**: Both similarity measures can be mixed with a linear combination (see Formula (3)).
  (3) Comb=αCos+(1−α)Lev,α∈[0,1]

In order to evaluate the proposed methods with the datasets described in this data paper, we calculate the Precision at rank n (P@n) based on 84 researcher variables and 170 candidate variables. This means that a relevant variable is proposed by our automatic methods at top n.

The obtained result summarized in Table 3 highlights good behavior of our method and encouraging results with lemmatization. Other results are given in [9] and in the following repository: https://github.com/bilson98/STAGE_Cirad.

**Table 3:** Results of Comb measure that combines Lev and Cos measures (P@n).

Rank (n)	α given the best result	Precision (without lemmatization)	Precision (with lemmatization)
1	0.3	42.9 %	44.0 %
3	0.2	54.8 %	55.9 %
5	0.3	63.1 %	64.3 %
10	0.3	71.4 %	73.8 %

## Ethics Statement

No conflict of interest exists in this submission. The authors declare that the work described in this paper is original and not under consideration for publication elsewhere, in whole or in part. Its publication is approved by all the authors listed.

## CRediT authorship contribution statement

**Sandrine Auzoux:** Methodology, Resources, Data curation, Writing – review & editing. **Billy Ngaba:** Methodology, Software, Resources, Data curation, Writing – review & editing. **Mathias Christina:** Resources, Data curation, Writing – review & editing. **Benjamin Heuclin:** Methodology, Resources, Data curation, Writing – review & editing. **Mathieu Roche:** Methodology, Writing – original draft.

## Declaration of Competing Interest

The authors declare that they have no financial or personal interests that could influence the work reported in this paper.

## Data Availability

Experimental dataset for mapping researcher variables from service plant trials to AEGIS dictionary variables (Original Data) (Dataverse).
